# Supplementary material for: Identification of photoperiod-induced specific miRNAs in the adrenal glands of Sunite sheep (Ovis aries)
Source: Front Vet Sci. 2022 Jul 22;9:888207. doi: 10.3389/fvets.2022.888207 (PMC9354845; doi:10.3389/fvets.2022.888207)
Supplement: Supplementary file 1 [file Data_Sheet_1.ZIP › Supplementary file/miRDeep2 file.zip!/miRDeep2 file/Novel_Structure/LP42A2/Novel_143.pdf]

Provisional ID	:	NC_019471.2_24593
Score total	:	52.3
Score for star read(s)	:	3.9
Score for read counts	:	45
Score for mfe	:	1.8
Score for randfold	:	1.6
Score for cons. seed	:	
Total read count	:	100
Mature read count	:	43
Loop read count	:	0
Star read count	:	57

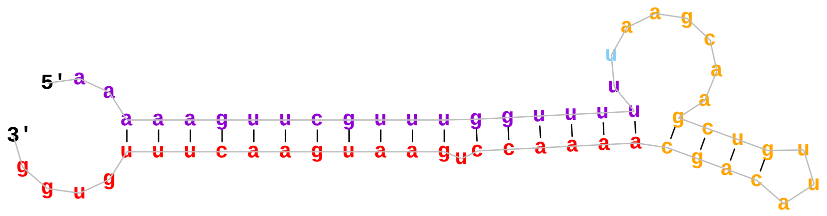

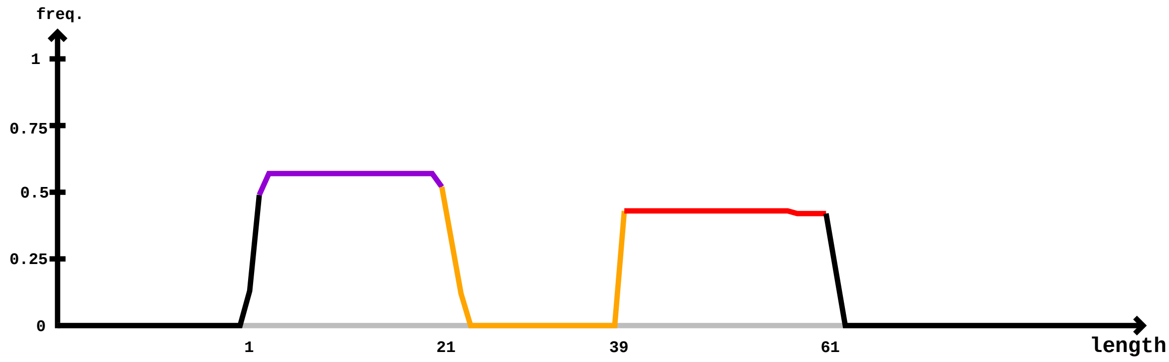

Star **Mature**

```
5'- auacacuacugggcuggccaaaaaguucguuugguuuuuuaagcaagcuguuacagcaaaaccugaaugaacuuuguggccaacccaagacucuguucccacaggcagaca   -3'    obs
    auacacuacugggcuggccaaaaaguucguuugguuuuuuaagcaagcuguuacagcaaaaccugaaugaacuuuguggccaacccaagacucuguucccacaggcagaca          exp
    ........((((.((((((.((((((((((((((((........((((...)))))))))).))))))))))).))))))).))))....((((((......))))))..   reads    mm    sample
    ..................caaaaaguucguuuggGuuuuu.......................................................................   13       1     seq
    ...................aaaaaguucguuuggGuuu.........................................................................   5        1     seq
    ...................aaaaaguucguuugguuuuC........................................................................   1        1     seq
    ...................aaaaaguucguuuggGuuuu........................................................................   24       1     seq
    ...................aaaaaguucguuugguuuuuu.......................................................................   2        0     seq
    ...................aaaaaguucguuuggGuuuuua......................................................................   4        1     seq
    ....................aaaaguucguuuggGuuuuua......................................................................   8        1     seq
    .........................................................aaaaccugaaugaacuuu....................................   1        0     seq
    .........................................................aaaaccugaaugaacuuuUugg................................   42       1     seq
```
